# Novel combinations for the treatment of metastatic breast cancer

**DOI:** 10.3390/cancers2010001

**Published:** 2010-01-14

**Authors:** Linda T. Vahdat

**Affiliations:** Weill Cornell Medical College, 425 East 61st Street, 8th Floor, New York, NY 10065, USA; E-Mail: ltv2001@med.cornell.edu; Tel.: +1-212-821-0644; Fax: +1-212-821-0758

**Keywords:** metastatic breast cancer, anthracycline failure, taxane failure, combination therapy, chemotherapy, targeted or biologic therapy, outcomes, survival

## Abstract

Anthracyclines and taxanes represent the mainstay of first-line cytotoxic therapy for metastatic breast cancer (MBC), but most patients eventually develop resistance to these agents. Consequently, alternative combinations for MBC therapy are the subject of much ongoing research. Capecitabine and ixabepilone is the only chemotherapy combination specifically approved for MBC after failure of anthracyclines and taxanes. Other options have limited data to support their use in this setting but are commonly used in practice. Future MBC therapies will likely combine alternative chemotherapies and novel biologic agents, and numerous ongoing trials should help to further define the proper use of these regimens.

## 1. Introduction

Breast cancer accounts for 26% of all diagnosed female cancers in the United States, and is the second most common cause of cancer mortality. Approximately 182,460 new cases and 40,480 deaths were anticipated in 2008 [1]. Only a small minority will have metastatic breast cancer (MBC) at the time of presentation (approximately 6%), but recurrence rates vary from 20% to 60% depending on molecular subtype, tumor size, and nodal status [2,3]. Despite therapeutic advances in recent years, prognosis remains poor for MBC, with a median survival of up to 4 years [4] and a 5-year survival rate of approximately 27% [2]. 

Treatment for MBC is rarely curative, so palliating symptoms, prolonging survival, and maintaining/improving quality of life are usually the main treatment goals. Less toxic therapies are therefore preferred, if possible. As the median age of breast cancer patients is 65 years, a substantial proportion of older patients may not tolerate highly aggressive therapy because of associated toxicity. There is currently no gold standard regimen for MBC, and treatment guidelines recommend several alternatives (Table 1) [5]. Improved survival in a randomized controlled trial is the standard requirement for drug approval first-line, but time-to-progression (TTP) and progression-free survival (PFS) have supported second- and third-line approvals [6]. 

**Table 1:** Preferred chemotherapy agents and regimens for MBC [5].

**Single agents**
Doxorubicin, epirubicin; pegylated liposomal doxorubicin, paclitaxel, docetaxel, nab-paclitaxel, capecitabine, vinorelbine, gemcitabine
**Combination chemotherapy**
CAF/FAC; FEC; AC; EC; AT; CMF; docetaxel + capecitabine; gemcitabine + docetaxel
**Combinations with molecular targeted therapies**
Trastuzumab – first-line, HER2-positive disease
Trastuzumab + paclitaxel ± carboplatin
Trastuzumab + docetaxel
Trastuzumab + vinorelbine
Trastuzumab + capecitabine
Trastuzumab-exposed, HER2-positive disease
Lapatinib + capecitabine
Trastuzumab + other first-line agents
Trastuzumab + capecitabine
Trastuzumab + lapatinib
Bevacizumab – first-line, HER2-negative disease
Bevacizumab + paclitaxel
**Other active options**
Cyclophosphamide, mitoxantrone, cisplatin; oral etoposide; vinblastine; fluorouracil continuous infusion; ixabepilone, capecitabine + ixabepilone

Abbreviations: AC, doxorubicin/cyclophosphamide; AT, doxorubicin + docetaxel or paclitaxel; CAF/FAC, cyclophosphamide, doxorubicin + fluorouracil; CMF, cyclophosphamide, methotrexate, fluorouracil; EC, epirubicin + cyclophosphamide; FEC, fluorouracil, epirubicin + cyclophosphamide.

Predominant factors affecting treatment choice include patient characteristics, such as age, menopausal status, and personal preference, and tumor and disease characteristics, such as site and extent of disease, tumor hormone receptor status, level of human epidermal growth factor receptor 2 (HER2) expression, prior treatment exposure, and disease-free interval [5].

Cytotoxic chemotherapies approved for MBC are myriad and include the anthracyclines (doxorubicin, epirubicin, and liposomal doxorubicin), the taxanes (paclitaxel, nab-paclitaxel, and docetaxel), capecitabine, gemcitabine, and an epothilone, ixabepilone. Additional active agents include cyclophosphamide, mitoxantrone, cisplatin, oral etoposide, vinorelbine, vinblastine, and 5-fluorouracil. Another agent showing promise for MBC is E7389 (eribulin), a synthetic analog of halichondrin B (HB) and nontaxane microtubule dynamics inhibitor, shown to have highly potent anticancer activity in vitro and in vivo [7]. In a phase II trial of patients with refractory breast cancer who had received 2–5 prior chemotherapy regimens and progressed on or within six months of their last chemotherapy (*N* = 269 for efficacy population), the overall response rate (ORR) with eribulin was 14% according to investigator assessment (IA) [8]. A phase III study of eribulin versus the physician’s treatment choice is underway in locally recurrent or MBC. Patients are eligible if previously treated with ≥2 and ≤5 prior chemotherapy regimens, including an anthracycline and taxane.

## 2. First-line therapy

Paclitaxel and docetaxel are microtubule stabilizers and among the most active agents in MBC. They are widely used in sequence or combination with an anthracycline first-line for locally-advanced disease or MBC. A recent meta-analysis, including trial data from >4,000 patients, showed increased ORRs and prolonged PFS with taxane regimens, but not prolonged overall survival (OS) versus nontaxane regimens [9]. Gemcitabine combined with paclitaxel is another approved first-line option for MBC, after failure of prior anthracycline-containing adjuvant chemotherapy [5]. Approval was based on a phase III trial of 529 patients, in which this combination produced higher ORRs (41% *vs.* 26%, *p* = 0.0002), a longer median TTP (6.1 months *vs.* 4.0 months, *p* = 0.0002), and a longer OS (18.6 months *vs.* 15.8 months, *p =* 0.049) than paclitaxel alone [10]. 

The first-line combination of gemcitabine plus nab-paclitaxel was recently evaluated in a phase II trial (*n* = 50). Nab-paclitaxel (125 mg/m^2^) plus gemcitabine (1,000 mg/m^2^) led to an ORR of 50%, and the median duration of response was 6.9 months. Median PFS was 7.9 months, but median OS was not reached. Therapy was well tolerated; neutropenia was the most common adverse event (grade 3, 43%; grade 4, 12%) [11]. 

Biologic targeted therapies are a major aspect of the treatment spectrum for MBC and have further improved prognosis. Trastuzumab, which targets HER2, may be used as monotherapy or combined with chemotherapies including paclitaxel, docetaxel, vinorelbine, or capecitabine for HER2-positive MBC [5]. Results from a recent randomized phase III trial found that immediate combination therapy with trastuzumab (4 mg/kg loading dose, followed by 2 mg/kg weekly) and docetaxel (60 mg/m^2^) was superior to sequential single-agent trastuzumab followed by the trastuzumab and docetaxel doublet after disease progression in terms of both PFS (HR, 4.24; *P* = 0.01) and OS (HR, 2.72; *P* = 0.04) [12]. While trastuzumab therapy is appropriate only for HER2-positive MBC, combining the antivascular endothelial growth factor (VEGF) monoclonal antibody bevacizumab and paclitaxel offers a first-line regimen for HER2-negative MBC [5,13]. Activity of bevacizumab is not limited to HER2-negative disease, however, as the combination of bevacizumab and trastuzumab appears to be active and well tolerated as first-line therapy for patients with HER2-positive disease (clinical benefit rate, 60%) [14]. Recent data from the RIBBON-2 trial suggest that bevacizumab combinations may also be useful beyond first-line therapy, as discussed in a subsequent section [15].

## 3. Recurrent or refractory disease

Established salvage cytotoxic agents frequently used after anthracyclines and taxanes include capecitabine, vinorelbine, gemcitabine, platinum salts, liposomal doxorubicin, and ixabepilone. Capecitabine and ixabepilone are the only chemotherapy agents approved for MBC after failure of anthracycline and taxane therapy [5]. Capecitabine monotherapy (1,250 mg/m^2^ twice daily for two weeks, in 3-week cycles) is approved for patients whose tumors are resistant to paclitaxel and an anthracycline, or paclitaxel alone where an anthracycline is contraindicated. In two randomized, phase II studies, capecitabine achieved ORRs in 20% to 27% of patients, with a median survival of approximately 1 year [16,17]. Capecitabine plus docetaxel (75 mg/m^2^ every three weeks) is indicated after prior anthracycline therapy on the basis of phase III trial results [18]. In addition, capecitabine is approved as salvage therapy in combination with the HER2-targeted agent lapatinib, in HER2-positive, taxane- and trastuzumab-pretreated disease. 

Ixabepilone is a microtubule inhibitor has a distinct mode of action to the taxanes and low susceptibility to tumor resistance mechanisms [19,20,21]. Ixabepilone is the first epothilone approved by the Food and Drug Administration (FDA) for locally-advanced or MBC, either as monotherapy following progression on an anthracycline, a taxane, and capecitabine, or combined with capecitabine following anthracycline and taxane failure. 

The clinical activity of ixabepilone monotherapy was shown in a phase II trial of 126 patients resistant to an anthracycline, a taxane, and capecitabine, with 88% receiving at least two lines of prior chemotherapy for MBC [22]. Single-agent ixabepilone produced ORRs in 12% of patients by independent radiology review (IRR), and 50% of patients achieved stable disease. The median duration of response was 5.7 months, and median PFS was 3.1 months. In 65 patients with MBC pretreated with an adjuvant anthracycline only, ixabepilone monotherapy 40 mg/m^2^ every three weeks led to an ORR of 42%, a median duration of response of 8.2 months and median OS of 22.0 months [23].

In a subsequent phase III trial of 752 patients, almost all (97%) had anthracycline- and/or taxane-resistant MBC. Anthracycline resistance was defined as disease recurrence occurring while on therapy or within 6 months of completing adjuvant or neoadjuvant therapy, or within three months of the last dose for MBC. Taxane resistance included progression while on therapy or within 12 months of the last dose in the adjuvant setting, or within 4 months in the metastatic setting. Capecitabine plus ixabepilone achieved an ORR of 35% versus 14% with capecitabine alone, according to IRR (*p* < 0.0001) [24,25]. The ORR by IA was 42% versus 23%, respectively. Median PFS was 5.7 months versus 4.1 months by IRR (*p* < 0.0001, 31% reduction in the risk of disease progression), and 5.3 months versus 3.3 months by IA (*p* = 0.0011, 22% risk reduction) [24,25,26]. These results of the pivotal trial are supported by a confirmatory phase III study of patients pretreated with an anthracycline and/or taxane (approximately 50% were resistant to taxanes) [26]. A subsequent pooled analysis of patients with taxane-resistant tumors in both trials showed an ORR rate of 39% with ixabepilone plus capecitabine versus 22% with capecitabine alone, a median PFS of 5.1 months versus 3.7 months and a median OS of 13.3 months versus 11.6 months, respectively [27].

Treatment options are particularly limited for patients with triple negative or basal-like MBC resistant to taxanes and/or anthracyclines [28], but ixabepilone plus capecitabine has shown efficacy in these patients. In a pooled subgroup analysis of the phase III trials [24,26], capecitabine plus ixabepilone achieved an ORR of 31% in 191 patients with triple-negative tumors (*vs.* 15% in 208 patients treated with capecitabine alone), and a median PFS of 4.2 months (*vs.* 1.7 months with capecitabine alone) [29]. Subgroup analyses of HER2-positive or ER-negative patients have shown that this combination is associated with higher ORRs than capecitabine monotherapy [30,31]. Ixabepilone also yielded clinical benefit in two other challenging subgroups of MBC: patients that relapse within 1 year of completing adjuvant anthracycline/taxane therapy [32] and symptomatic patients (Karnofsky performance status 70–80) [33].

A randomized phase III adjuvant trial sponsored by the Sarah Cannon Research Institute and Bristol-Myers Squibb (TITAN; NCT00789581) is currently enrolling patients with triple-negative breast cancer. Patients will receive doxorubicin plus cyclophosphamide followed by ixabepilone versus paclitaxel (TITAN). A second phase III adjuvant trial (PACS 08; NCT00630032) is examining the role of sequential 5-fluorouracil, epirubicin, and cyclophosphamide (FEC-100), and ixabepilone (*vs.* FEC-100 and docetaxel) for triple-negative breast cancer. The planned accrual is 2,500 patients.

Various novel chemotherapy combinations and head-to-head studies with ixabepilone are under investigation. A phase II trial (IXTEND) is recruiting to compare ixabepilone plus capecitabine with docetaxel plus capecitabine in MBC (NCT00546364; www.clinicaltrials.gov). Ongoing phase II studies of patients with MBC also are evaluating ixabepilone in combination with liposomal doxorubicin and carboplatin. Preliminary data from a phase Ib study indicate that the combination of ixabepilone with epirubicin has a favorable toxicity and efficacy profile [34]. This study has now been completed.

### Novel chemotherapy and targeted agent combinations

The future of MBC therapy will focus on combinations incorporating effective chemotherapy and novel biologics. This approach has yielded notable success with the HER2-targeted agents trastuzumab and lapatinib, and some success with the angiogenesis inhibitor bevacizumab. All of these agents have improved TTP and/or PFS compared with chemotherapy alone in patients with MBC. Several trials are evaluating combinations of these drugs and other targeted therapies with chemotherapy in the first-line MBC setting. Phase III trial results are summarized in Table 2 [13,35,36,37,38,39,40,41,42,43,44].

## 4. Bevacizumab combinations

In the pivotal, first-line, phase III study in HER2-negative MBC, adding bevacizumab 10 mg/kg to paclitaxel 90 mg/m^2^ significantly increased the ORR (37% *vs.* 21%, *p* < 0.001) and PFS (11.8 months *vs.* 5.9 months; *p* < 0.001) but OS was similar in both treatment groups [13]. Clinical experience has suggested that bevacizumab plus nab-paclitaxel is effective and well tolerated as a first-line MBC therapy (Link *et al.* [45]: retrospective analysis of 40 women; Lobo *et al.* [46]: single institution experience of six patients receiving bi-weekly nab-paclitaxel 100 mg/m^2^ plus bevacizumab 10 mg/kg, three patients also received gemcitabine 1,000 mg/m^2^). Early results from a phase II trial showed a 91% ORR (10/11 patients) with bevacizumab 10 mg/kg plus gemcitabine 1,500 mg/m^2^ and nab-paclitaxel 150 mg/m^2^, administered on days 1 and 15 of 28-day cycles [47].

**Table 2:** Summary of phase III clinical trials combining chemotherapy and targeted biologic therapy for the first-line treatment of MBC.

Reference	Chemotherapy	N	ORR (%)	TTP (months)	PFS (months)	OS (months)
**Bevacizumab combinations**						
Miller *et al.* 2007 [13]	Paclitaxel + bevacizumab	368	37		11.8	26.7
	Paclitaxel	354	21		5.9	25.2
Miles *et al.* 2008 [35]	Docetaxel + bevacizumab (7.5 mg/kg)	248	55		Improved versus docetaxel (*p* = 0.0035)	
	Docetaxel + bevacizumab (15 mg/kg)	247	63		Improved versus docetaxel (*p* < 0.0001)	
	Docetaxel	241	44			
Miller *et al.* 2005 [36]	Capecitabine + bevacizumab	232	20		4.9	15.1
	Capecitabine	230	9		4.2	14.5
Robert *et al.* 2009 [37]	Capecitabine + bevacizumab	409	35		8.6	29.0
	Capecitabine	206	24		5.7	21.2
	Taxane/anthracycline + bevacizumab	415	51		9.2	25.2
	Taxane/anthracycline	207	38		8.0	23.8
**Trastuzumab combinations**						
Slamon *et al.* 2001 [38]	Paclitaxel + trastuzumab	92	41	6.9		22.1
	Paclitaxel	96	17	3.0		18.4
Robert *et al.* 2006 [39]	Paclitaxel + carboplatin + trastuzumab	98	52		10.7	35.7
	Paclitaxel + trastuzumab	98	36		7.1	32.2
Pegram *et al.* 2007 [40]	Docetaxel + carboplatin + trastuzumab	131	73	10.4		36.6
	Docetaxel + trastuzumab	131	73	11.1		36.4
Von Minckwitz *et al.* 2008 [41]	Capecitabine + trastuzumab	78	49		8.5	20.3
	Capecitabine	78	25		5.6	19.9
**Lapatinib combinations**						
Cameron *et al.* 2008 [42]	Capecitabine + lapatinib	198	24	6.2		15.6
	Capecitabine	201	14	4.3		15.3
Di Leo *et al.* 2008 [43]	Paclitaxel + lapatinib	291	35	29.0 weeks		99.1 weeks
	Paclitaxel	288	25	22.9 weeks		87.0 weeks
**Trastuzumab and lapatinib combination**						
O’Shaughnessy *et al.* 2008 [44]	Trastuzumab + lapatinib	148	10	3 (12 weeks)		51.6
	Lapatinib	148	7	2 (8.4 weeks)		39.0

Adding bevacizumab to docetaxel first-line significantly improved clinical outcomes in MBC in the phase III AVADO trial [35], although gains were modest for TTP. Preliminary data showed an increased ORR with bevacizumab 7.5 mg/kg or 15 mg/kg combined with docetaxel 100 mg/m^2^ every three weeks (55% and 63% compared with 44% for single-agent docetaxel). The stratified hazard ratio (HR) for PFS was reduced with the addition of bevacizumab at both doses compared with docetaxel alone (0.69 and 0.61 for bevacizumab 7.5 mg/kg and 15 mg/kg, respectively). OS data has not been reported. A phase III trial will evaluate adding bevacizumab to docetaxel and trastuzumab in first-line, HER2-positive MBC (NCT00391092).

In a phase III trial of patients with heavily-pretreated MBC, capecitabine 2,500 mg/m^2^/day on days 1 to 14 every three weeks, plus bevacizumab 15 mg/kg on day 1, significantly increased the ORR (20% *vs.* 9%; *p* = 0.001), but not PFS (4.9 months *vs.* 4.2 months), or OS (15.1 months *vs.* 14.5 months) [36]. A single-arm, phase II study of capecitabine plus bevacizumab in 106 patients with chemo-naïve MBC reported an ORR of 38% [48]. A phase III trial will compare capecitabine and paclitaxel, both combined with bevacizumab, as first-line treatment for MBC (NCT00600340, not yet recruiting).

Two ongoing phase III RIBBON trials further elucidated the role of bevacizumab combinations. Results of RIBBON 1, which combined bevacizumab with chemotherapy in previously-untreated MBC (NCT00262067), provided confirmation that incorporating bevacizumab 15 mg/kg into 28-day chemotherapy regimens with a taxane (nab-paclitaxel 260 mg/m^2^ or docetaxel 75 or 100 mg/m^2^ on day 1), capecitabine-(2,000 mg/m^2^/day on days 1 to 14), or anthracycline-based therapy (day 1) significantly improved PFS over chemotherapy alone (Table 2) [37]. The RIBBON 2 trial combined bevacizumab with chemotherapy (docetaxel, gemcitabine, or vinorelbine) in previously-treated MBC and, in contrast to the previous study reported above, identified a significant improvement in PFS over chemotherapy alone (7.2 months *vs.* 5.1 months) [15]. The upcoming RIBBON-3 trial will explore the efficacy of continuing bevacizumab through multiple lines of chemotherapy.

Irinotecan, a topoisomerase I inhibitor, was originally reported to be of limited utility for salvage therapy of breast cancer [49], but is currently being re-evaluated in this setting in phase II trials. One of these adds bevacizumab to twice-weekly irinotecan in patients with MBC and brain metastases (NCT00476827).

## 5. Trastuzumab combinations

Current guideline-preferred cytotoxics for combination with trastuzumab include paclitaxel, both with and without carboplatin (FDA approved), docetaxel, and vinorelbine (Table 1). In a phase III trial, adding a loading dose of 4 mg/kg trastuzumab, followed by a weekly 2 mg/kg dose, to 3-weekly paclitaxel (175 mg/m^2^) first-line for HER2- positive, locally-advanced, or MBC prolonged TTP (6.9 months compared with 3.0 months for paclitaxel alone, *p* < 0.001) [38]. In a subsequent phase II study, adding trastuzumab to weekly paclitaxel prolonged TTP (301 days *vs.* 204 days for paclitaxel alone) [50]. Similarly, adding trastuzumab to docetaxel in a phase II trial first-line significantly improved TTP compared with docetaxel alone (11.7 months *vs.* 6.1 months) [51].

Chemotherapy doublets with trastuzumab first-line have also been evaluated. A phase III trial comparing trastuzumab 4 mg/kg loading dose plus 2 mg/kg weekly and paclitaxel 175 mg/m^2^ every three weeks, with trastuzumab plus paclitaxel at the same doses combined with carboplatin area-under-the-curve (AUC) 6 every three weeks.The ORR was 36% versus 52% (*p* = 0.04), respectively, with a median PFS of 7.1 and 10.7 months (*p* = 0.03). OS was similar between groups at 32.2 and 35.7 months (*p* = 0.76) [39]. Results from the phase III BCIRG 007 trial were reported at The American Society of Clinical Oncology (ASCO) Annual Meeting in 2007 [40], showing that trastuzumab (4 mg/kg loading dose, 2 mg/kg weekly, then 6 mg/kg 3-weekly) combined with docetaxel (100 mg/m^2^) and carboplatin (AUC 6), or trastuzumab plus docetaxel without carboplatin were highly effective as first-line therapy for HER2-positive MBC, achieving median OS times in excess of 36 months. There was no significant difference between arms in median TTP (10.4 months *vs.* 11.1 months respectively, *p* = 0.57) or ORR (73% in both arms). These combinations are being evaluated first-line in an ongoing phase III trial (NCT00047255). 

Many other chemotherapy agents are being combined with trastuzumab in phase III trials. For example, a phase III trial is planned to compare docetaxel and vinorelbine, both in combination with trastuzumab, in chemo-naïve patients with HER2-positive MBC (recruiting at present; NCT00430001). Trastuzumab plus capecitabine is being assessed in patients with MBC progressing during trastuzumab therapy [41]. Preliminary results indicate that continuing trastuzumab after progression and adding capecitabine is more effective than second-line capecitabine alone (PFS 8.5 months with 48 events *vs.* 5.6 months with 53 events). In another ongoing phase III trial (NCT00440622), capecitabine and gemcitabine, both combined with trastuzumab, are being compared in patients with pretreated MBC. Trastuzumab plus the semisynthetic taxane, larotaxel, is currently undergoing phase II evaluation in MBC (NCT00387907).

Combining trastuzumab with other targeted agents may also have promise in MBC. For example, a phase II trial is examining trastuzumab plus lapatinib as first-, second-, or third-line therapy for HER2-positive disease (NCT00470704). A phase II study has already shown that trastuzumab as a 4 mg/kg loading dose followed by 2 mg/kg/weekly, or an 8 mg/kg loading dose followed by 6 mg/kg/3-weekly, plus a pertuzumab 840 mg loading dose followed by 420 mg 3-weekly, produced an ORR of 24% (16/66 patients), with 50% of patients achieving clinical benefit (including stable disease) [52]. A phase III first-line MBC trial of this regimen is due to complete in 2012 (CLEOPATRA; NCT00567190).

## 6. Lapatinib combinations

A novel treatment regimen that has recently been approved by the FDA is capecitabine plus lapatinib, for HER2-positive MBC following prior anthracyclines, taxanes, and trastuzumab. Approval was based on a phase III trial of heavily pretreated and trastuzumab-refractory patients (*N* = 399). Adding lapatinib 1,250 mg/day to capecitabine 2,000 mg/m^2^ on days 1 to 14 of a 21-day cycle increased median TTP from 4.3 months to 6.2 months (*p* < 0.001), with a trend toward improved OS (15.6 months *vs.* 15.3 months; HR: 0.78, 95% confidence interval [CI]: 0.55–1.12; *p* = 0.177) versus capecitabine 2,500 mg/m^2^ [42]. There were also fewer cases with central nervous system involvement at progression in the combination group (4 *vs.* 13; *p* = 0.045).

A phase III study has investigated lapatinib 1,500 mg/day plus paclitaxel 175 mg/m^2^ versus paclitaxel monotherapy for MBC first-line [43]. In the intent-to-treat population of 579 patients, no significant differences between groups were demonstrated for median duration of response (28 weeks *vs.* 27 weeks) or TTP (29 weeks *vs.* 23 weeks). The ORR was 35% versus 25% (*p* = 0.008). In 86 HER2-positive patients (15%), paclitaxel plus lapatinib significantly improved TTP, event-free survival, ORR and clinical benefit rate compared with paclitaxel plus placebo. No significant differences between treatment groups were observed for any endpoint in HER2-negative patients.

Other lapatinib combinations with chemotherapy, hormonal therapy, or targeted agents are being tested. A phase II single-arm study (EGF105764) has evaluated lapatinib 1,500 mg/day and paclitaxel 80 mg/m^2^ weekly every three weeks in 57 patients with newly diagnosed, HER2-positive MBC. The ORR was 51% and the median duration of response was 40 weeks [53]. Phase II trials of lapatinib plus vinorelbine (NCT00709618, NCT00754702) or nab-paclitaxel (NCT00709761) first- or second-line for MBC are recruiting. In a randomized trial of 1,286 postmenopausal women with hormone-receptor-positive untreated MBC from 212 international centers (EGF30008), patients received once-daily lapatinib 1,500 mg plus letrozole 2.5 mg versus letrozole alone [54]. In the HER2-positive population, median PFS significantly increased from 3.0 months in the letrozole group to 8.2 months in the lapatinib plus letrozole group (*p* = 0.019), and the ORR was 15% versus 28% (*p* = 0.021). In a phase II trial (*n* = 52), preliminary data for 32 patients receiving lapatinib 1,500 mg/day plus bevacizumab 10 mg/kg every two weeks has shown a crude 12-week PFS rate of 63% and an ORR of 13% [55]. The lapatinib plus trastuzumab combination is also under clinical investigation, as mentioned previously.

## 7. Ixabepilone and targeted therapy combinations

The established antitumor activity of ixabepilone in MBC has prompted investigations of its efficacy combined with chemotherapy, or targeted therapies including trastuzumab, lapatinib, bevacizumab, and cetuximab. Preclinically, ixabepilone and trastuzumab have synergistic antitumor activity [56]. In a phase II trial of 39 patients with HER2-positive MBC, cohort 1 had received no prior chemotherapy or trastuzumab for MBC (*n* = 15) and cohort 2 had received one or two trastuzumab-containing regimens (*n* = 24). Both cohorts received ixabepilone 40 mg/m^2^ every three weeks plus a trastuzumab 8 mg/kg loading dose on day 1 followed by 6 mg/kg every three weeks. The median age of patients was 51 and a median of seven cycles of therapy were administered in each cohort. Ixabepilone plus trastuzumab produced an ORR of 51% (clinical benefit rate 56%) and median age a median TTP of 6.1 months [57].

Phase II data indicate that ixabepilone, trastuzumab and carboplatin is active with acceptable toxicity first-line for HER2-positive MBC [58]. Of the 57 patients evaluated for response, two achieved a complete response (3.5%), 22 had partial response (38.6%) and 13 had stable disease extending beyond 6 months (22.8%). The median PFS was 8 months (95% CI: 6.0–9.7). Three phase II studies (ongoing or completed) combine trastuzumab and ixabepilone as first- or second-line therapy for HER2-positive advanced or MBC (NCT00490646, NCT00079326, NCT00337649). 

Ixabepilone has antiangiogenic properties [59] and synergistic activity when used in combination with the antiangiogenic agents bevacizumab and sunitinib in a range of human tumor xenograft models, including breast [60,61]. Notably, the synergistic activity shown with ixabepilone was greater than with paclitaxel plus antiangiogenic agents, which may be, at least in part, attributable to the reduced susceptibility of ixabepilone for drug efflux pumps. A preliminary report illustrates that in combination with bevacizumab, both the weekly and every-3-week schedules of ixabepilone demonstrated activity that was at least comparable to paclitaxel plus bevacizumab [62]. In addition, a randomized phase III trial is comparing paclitaxel to nab-paclitaxel or ixabepilone with all arms in combination with bevacizumab (CALGB 40452).

## 8. Combinations with PARP Inhibitors

Patients whose tumors are HER2-negative, particularly those with triple-negative disease, may experience significant benefits from members of a novel class of small molecules that inhibit poly(adenosine diphosphate-ribose) polymerase (PARP). PARP inhibitors block DNA damage repair, and their mechanism of action is thought to complement the DNA-damaging effects of platinum agents and antimetabolites [63]. One such compound, BSI-201, significantly improved the PFS (6.9 *vs.* 3.3; HR, 0.34 [0.20–0.58]; *P* < 0.0001) and OS (9.2 *vs.* 5.7; HR, 0.35 [0.19–0.65]; *P* = 0.0005) of gemcitabine and carboplatin in patients with triple-negative MBC in a recent randomized phase II study [63]. Another PARP inhibitor, olaparib, produced a 38% response rate in pretreated BRCA-deficient advanced breast cancer tumors [64].

## 9. Other investigational targeted therapy regimens

Several phase III studies of other regimens combining cytotoxic chemotherapy and targeted therapy are current or planned. Novel HER2 inhibitors beyond lapatinib are currently undergoing investigation in HER2-positive breast tumors, typically those which have progressed on or after trastuzumab therapy. Pertuzumab, which inhibits HER2 using a different mechanism than trastuzumab, has shown promise in combination with trastuzumab, even when disease has progressed on either agent alone [65]. Pertuzumab is also being evaluated in combination with trastuzumab and docetaxel first-line for MBC (NCT00567190). Likewise, in phase I/II trials, the pan-HER inhibitor neratinib has demonstrated clinical activity in combination with several chemotherapeutic agents, including paclitaxel [66], capecitabine [67], and vinorelbine [68].

Because mutations in the PI3-kinase/Akt pathway have been linked to trastuzumab resistance, researchers have pursued the downstream effector mammalian target of rapamycin (mTOR) in attempts to bypass such resistance. Dose-finding trials have revealed encouraging efficacy for the mTOR inhibitor everolimus (RAD001) in several trastuzumab-containing chemotherapy regimens, including those with paclitaxel [69], docetaxel [70], and vinorelbine [71], and in non-trastuzumab-containing combinations with capecitabine [72] and cisplatin/paclitaxel [73]. 

Sunitinib is an oral, small-molecule, receptor tyrosine kinase (RTK) inhibitor that is the current standard of care for renal cell carcinoma and gastrointestinal stromal tumor. Sunitinib inhibits cellular signaling by targeting multiple RTKs, including platelet-derived growth factor receptor (PDGF-R) and vascular endothelial growth factor receptors (VEGF-R), which play a role in both tumor angiogenesis and tumor cell proliferation. In a phase II study of 64 patients with MBC pretreated with an anthracycline and a taxane, sunitinib was associated with an ORR of 11% [74]. Final results from a randomized phase II study (SABRE-B) showed that the addition of sunitinib to the approved paclitaxel plus bevacizumab regimen first-line was poorly tolerated, and the study was closed prematurely [75]. In addition, the results of the SUCON trial failed to demonstrate that sunitinib delays tumor progression following an objective response to taxane therapy [76]. 

In contrast, in patients with HER2-negative MBC, the multi-RTK inhibitor sorafenib showed promise in combination with capecitabine in the form of significantly improved PFS (6.4 months *vs.* 4.1 months; HR, 0.576 [0.410–0.809]) [77]. Sorafenib is now being combined with paclitaxel (NCT00499525), vinorelbine (NCT00764972), ixabepilone (NCT00825734), nab-paclitaxel (NCT00607438) and several hormonal therapies in ongoing phase II trials. The benefits of the anti-VEGFR2 monoclonal antibody IMC-1121B are also being explored in a phase III trial comparing single-agent docetaxel and docetaxel plus IMC-1121B in more than 1,100 patients with previously untreated HER2-negative MBC (NCT00703326). 

## 9. Conclusions

In locally-advanced breast cancer and MBC, combination therapy with cytotoxic agents and targeted biologic agents has afforded significant clinical benefits in both first-line and salvage treatment settings, including improved tumor response and prolonged TTP compared with chemotherapy alone. This enhanced activity is largely attributable to additive or synergistic activity and generally nonoverlapping toxicity profiles for targeted and cytotoxic therapies. The results of numerous ongoing clinical trials combining cytotoxics and/or targeted biologic agents are awaited with interest, as it is anticipated that these will help to define the optimal role of various regimens in the management of MBC across multiple lines of therapy.
